# Supplementary material for: Targeting IRE1α improves insulin sensitivity and thermogenesis and suppresses metabolically active adipose tissue macrophages in male obese mice
Source: bioRxiv. 2025 Jan 30:2024.07.17.603931. Originally published 2024 Jul 19. Preprint. [Version 3] doi: 10.1101/2024.07.17.603931 (PMC11275733; doi:10.1101/2024.07.17.603931)
Supplement: Supplement 2 [file NIHPP2024.07.17.603931v3-supplement-2.pdf]

bioRxiv preprint doi: https://doi.org/10.1101/2024.07.17.603931; this version posted January 30, 2025. The copyright holder for this preprint (which was not certified by peer review) is the author/funder, who has granted bioRxiv a license to display the preprint in perpetuity. It is made available under aCC-BY 4.0 International license.

Supplemental Table 3

Primers used in this work

Gene	Accession Number	Forward Primer	Reverse Primer
Gapdh	NM_008084	AAAGACTGGAGCCCCACACTCTAC	ATCCCGTATTTCACCTCTGCTTC
Xbp1s	NM_013842	GAG TCC GCA GCA GGT	GTG TCA GAG TCC ATG
Xbp1u	NM_013842	CGCAGACTGCTCGAGATAGA	CCACAAGGCCGTGAGTTTTC
Chop	NM_007837	GCAGCGACAGAGCCAGAATA	CAAGGTGAAAGGCAGGGACT
Bip	NM_022310	GTGTGTGAGACCAGAACCGT	GCAGTCAGGCAGGAGTCTTA
Edem1	NM_026041	CAAGTGTGGGTACGCCACG	AAAGAAGCTCTCCATCCGGTC
Blos1	NM_013886	CAAGGAGCTGCAGGAGAAGA	CCAGGAGGGTGAAGTAAGAGG
Col61a	NM_009933	TGCTCAACATGAAGCAGACC	TTGAGGGAGAAAGCTCTGGA
Atf4	NM_009716	CCTATAAAGGCTTGCGGCCA	GCTGGATTTCGTGAAGAGCG
Ucp1	NM_009463	CACCTTCCCCCTGGACACT	CCCTAGGACACCTTTATACCT
Prdm16	NM_001277052	AGGAGGAGGAGAGAGATTCCG	GTCCGGGTCAGGTTCATACAT
Pgc1-α	NM_008904	CCCTGCCATTGTTAAGACC	TGCTGCTGTTCCTGTTTTC
Cidea	NM_009369	ATCACAACTGGCCTGGTTACG	TACTACCCGGTGTCCATTTCT
Cox5b	NM_007752	TGCTACCTCCAAAGGCAGCTTC	CATCGCTGACTCTCGCCTTTGT
Cox7a1	NM_022025	CAGCGTCATGGTCAGTCTGT	AGAAAACCGTGTGGCAGAGA
Cox8b	NM_009944	GAACCATGAAGCCAACGACT	GCGAAGTTCACAGTGGTTCC
Ppara	NM_011144	TTCCCTGTGAACTGACGTTT	CCACCATGTTGGATGGATGTG
Dio2	NM_010050	AGAGTGGAGGCGCATGCT	GGCATCTAGGAGGAAGCTGTT
B1ar	NM_007419	CCGAAAGCAGGTGAATGCAA	AGCCAGTAAGCCATACTAAGCCACA
B2ar	NM_009715	CATTGATGTGTTGTGCGTCA	ACTCGGGCCTTATTCTTGGT
B3ar	NM_007420	CCTTCCGTCGTCTTCTGTGT	AGCCATCAAACCTGTTGAGC
Tnf-α	NM_013693	CTGAACTTCGGGGTGATCGG	CTACGACGTGGGCTACAGG
Il-1β	NM_008361	TGCCACCTTTTGACAGTGATG	TGATGTGCTGCTGCGAGATT
Il-6	NM_031168	CACTTCACAAGTCGGAGGCT	CTGCAAGTGCATCATCGTTGT
Mcp-1	NM_011333	AGGTCCCTGTCATGCTTCTG	TCTCCAGCCTACTCATTGGGA
Mgl1	NM_021357	CAGATCTGGGGCCGTCAAG	GGGAGGAAATGCATCTGGGT
Il-10	NM_010548	AGGCGCTGTCATCGATTTCT	ATGGCCTTGTAGACACCTTGG
F4/80	NM_010130	TCTGCTTCTGTACAGCCACG	CCTCAGAACCCACAGTGTCC
Cd68	NM_009853	TGTTCAGCTCCAAGCCCAAA	GTACCGTCACAACCTCCCTG
Abca1	NM_013454	TAGCAGCACCGTGTCTTGTC	GCGTGTCACTTTCATGGTCG
Cd36	NM_001159558	TCCAGCCAATGCCTTTGC	TGGAGATTACTTTTCAGTGCAGAA
Plin2	NM_007948	TCTGCGGCCATGACAAGTG	GCAGGCATAGGTATTGGCAAC
Atp6v1b2	NM_025989	AGCCTCGTCTCACCTACAAGA	CTCAGCGTATCTGGGAAACTT
Atp6v0d2	NM_001289445	AGCCAGCCTAACTCAGC	GCTTCTTCCTCATCTCCGTGTC
Lamp2	NM_010685	ATGTGCCTCTCTCCGGTTAAA	GCAAGTACCCTTTGAATCTGTCA

bioRxiv preprint doi: https://doi.org/10.1101/2024.07.17.603931; this version posted January 30, 2025. The copyright holder for this preprint (which was not certified by peer review) is the author/funder, who has granted bioRxiv a license to display the preprint in perpetuity. It is made available under aCC-BY 4.0 International license.

Lipa	NM_008519	GGAAACAGCAGAGGAAACACCT	CACGGGAGCCAAGACTAAAAC
Fabp4	NM_024406	GGGGCCAGGCTTCTATTCC	GGAGCTGGGTTAGGTATGGG
Fabp5	NM_001272613	ACGGCTTTGAGGAGTACATGA	CTCGGTTTTGACCGTGATG
Ctsb	NM_007798	CAATGGCCGTTGAATGCACA	TGGTGTATGGTAAGCAGTG
Trem2	NM_031254	AAAGCTAGCATGGAACCTCTC	TTGAATTCGAGCTCTCTAGACGT
Cd9	NM_007657	TGGGGCTATACCCACAAGGA	GCTTTGAGTGTTTCCCGCTG
Pepck	NM_011044	TGAAAGGCCGCACCATGTAT	GGGCGAGTCTGTCAGTTCAA
G6p	NM_008061	ATCCGGGGCATCTACAATG	TGGCAAAGGGTGTAGTGTCA
Acc	NM_133360	GCCTCTTCCTGACAAACGAG	TGACTGCCGAAACATCTCTG
Scd-1	NM_009127	GAGGCCTGTACGGGATCA	GCCCAGTCGTACACGTCA
Fasn	NM_007988	GCTGGCATTCGTGATGGAGTCGT	AGGCCACCAGTGATGATGTAACTCT
