# Supplementary material for: Targeting IRE1α improves insulin sensitivity and thermogenesis and suppresses metabolically active adipose tissue macrophages in obesity
Source: bioRxiv. 2024 Jul 19:2024.07.17.603931. Preprint. [Version 1] doi: 10.1101/2024.07.17.603931 (PMC11275733; doi:10.1101/2024.07.17.603931)
Supplement: Supplementary file 1 [file NIHPP2024.07.17.603931v1-supplement-1.pdf]

bioRxiv preprint doi: https://doi.org/10.1101/2024.07.17.603931; this version posted July 19, 2024. The copyright holder for this preprint (which was not certified by peer review) is the author/funder, who has granted bioRxiv a license to display the preprint in perpetuity. It is made available under aCC-BY 4.0 International license.

Supplemental Table 3

Primers used in this work

Gene	Forward Primer	Reverse Primer

bioRxiv preprint doi: https://doi.org/10.1101/2024.07.17.603931; this version posted July 19, 2024. The copyright holder for this preprint (which was not certified by peer review) is the author/funder, who has granted bioRxiv a license to display the preprint in perpetuity. It is made available under aCC-BY 4.0 International license.

**GAPDH**	AAAGACTGGAGCCCCACACTCTAC	ATCCCGTATTTCACCTCTGCTTC
**XBP1s**	GAG TCC GCA GCA GGT	GTG TCA GAG TCC ATG
**XBP1u**	CGCAGACTGCTCGAGATAGA	CCACAAGGCCGTGAGTTTTC
**Chop**	GCAGCGACAGAGCCAGAATA	CAAGGTGAAAGGCAGGGACT
**Bip**	GTGTGTGAGACCAGAACCGT	GCAGTCAGGCAGGAGTCTTA
**EDEM1**	CAAGTGTGGGTACGCCACG	AAAGAAGCTCTCCATCCGGTC
**Blos1**	CAAGGAGCTGCAGGAGAAGA	CCAGGAGGGTGAAGTAAGAGG
**Col61a**	TGCTCAACATGAAGCAGACC	TTGAGGGAGAAAGCTCTGGA
**ATF4**	CCTATAAAGGCTTGCGGCCA	GCTGGATTTCGTGAAGAGCG
**UCP1**	CACCTTCCCCCTGGACACT	CCCTAGGACACCTTTATACCT
**Prdm16**	AGGAGGAGGAGAGAGATTCCG	GTCCGGGTCAGGTTCATACAT
**PGC1-α**	CCCTGCCATTGTTAAGACC	TGCTGCTGTTCCTGTTTTC
**Cidea**	ATCACAACTGGCCTGGTTACG	TACTACCCGGTGTCCATTTCT
**Cox5b**	TGCTACCTCCAAAGGCAGCTTC	CATCGCTGACTCTCGCCTTTGT
**Cox7a1**	CAGCGTCATGGTCAGTCTGT	AGAAAACCGTGTGGCAGAGA
**Cox8b**	GAACCATGAAGCCAACGACT	GCGAAGTTCACAGTGGTTCC
**Ppara**	TTCCCTGTGAACTGACGTTT	CCACCATGTTGGATGGATGTG
**DIO2**	AGAGTGGAGGCGCATGCT	GGCATCTAGGAGGAAGCTGTT
**β1AR**	CCGAAAGCAGGTGAATGCAA	AGCCAGTAAGCCATACTAAGCCACA
**β2AR**	CATTGATGTGTTGTGCGTCA	ACTCGGGCCTTATTCTTGGT
**β3AR**	CCTTCCGTCGTCTTCTGTGT	AGCCATCAAACCTGTTGAGC
**TNF-α**	CTGAACTTCGGGGTGATCGG	CTACGACGTGGGCTACAGG
**IL-1β**	TGCCACCTTTTGACAGTGATG	TGATGTGCTGCTGCGAGATT
**IL-6**	CACTTCACAAGTCGGAGGCT	CTGCAAGTGCATCATCGTTGT
**MCP-1**	AGGTCCCTGTCATGCTTCTG	TCTCCAGCCTACTCATTGGGA

bioRxiv preprint doi: https://doi.org/10.1101/2024.07.17.603931; this version posted July 19, 2024. The copyright holder for this preprint (which was not certified by peer review) is the author/funder, who has granted bioRxiv a license to display the preprint in perpetuity. It is made available under aCC-BY 4.0 International license.

**Mgl1**	CAGATCTGGGGCCGTCAAG	GGGAGGAAATGCATCTGGGT
**IL-10**	AGGCGCTGTCATCGATTTCT	ATGGCCTTGTAGACACCTTGG
**F4/80**	TCTGCTTCTGTACAGCCACG	CCTCAGAACCCACAGTGTCC
**CD68**	TGTTCAGCTCCAAGCCCAAA	GTACCGTCACAACCTCCCTG
**Abca1**	TAGCAGCACCGTGTCTTGTC	GCGTGTCACTTTCATGGTCG
**CD36**	TCCAGCCAATGCCTTTGC	TGGAGATTACTTTTCAGTGCAGAA
**Plin2**	TCTGCGGCCATGACAAGTG	GCAGGCATAGGTATTGGCAAC
**ATP6V1B2**	AGCCTCGTCTCACCTACAAGA	CTCAGCGTATCTGGGAAACTT
**ATP6V0D2**	AGCCAGCCTAACTCAGC	GCTTCTTCCTCATCTCCGTGTC
**Lamp2**	ATGTGCCTCTCTCCGGTTAAA	GCAAGTACCCTTTGAATCTGTCA
**Lipa**	GGAAACAGCAGAGGAAACACCT	CACGGGAGCCAAGACTAAAAC
**Fabp4**	GGGGCCAGGCTTCTATTCC	GGAGCTGGGTTAGGTATGGG
**Fabp5**	ACGGCTTTGAGGAGTACATGA	CTCGGTTTTGACCGTGATG
**Ctsb**	CAATGGCCGTTGAATGCACA	TGGTGTATGGTAAGCAGTG
**Trem2**	AAAGCTAGCATGGAACCTCTC	TTGAATTCGAGCTCTCTAGACGT
**CD9**	TGGGGCTATACCCACAAGGA	GCTTTGAGTGTTTCCCGCTG
**PEPCK**	TGAAAGGCCGCACCATGTAT	GGGCGAGTCTGTCAGTTCAA
**G6P**	ATCCGGGGCATCTACAATG	TGGCAAAGGGTGTAGTGTCA
**ACC**	GCCTCTTCCTGACAAACGAG	TGACTGCCGAAACATCTCTG
**SCD-1**	GAGGCCTGTACGGGATCA	GCCCAGTCGTACACGTCA
**Fasn**	GCTGGCATTCGTGATGGAGTCGT	AGGCCACCAGTGATGATGTAACTCT

bioRxiv preprint doi: https://doi.org/10.1101/2024.07.17.603931; this version posted July 19, 2024. The copyright holder for this preprint (which was not certified by peer review) is the author/funder, who has granted bioRxiv a license to display the preprint in perpetuity. It is made available under aCC-BY 4.0 International license.

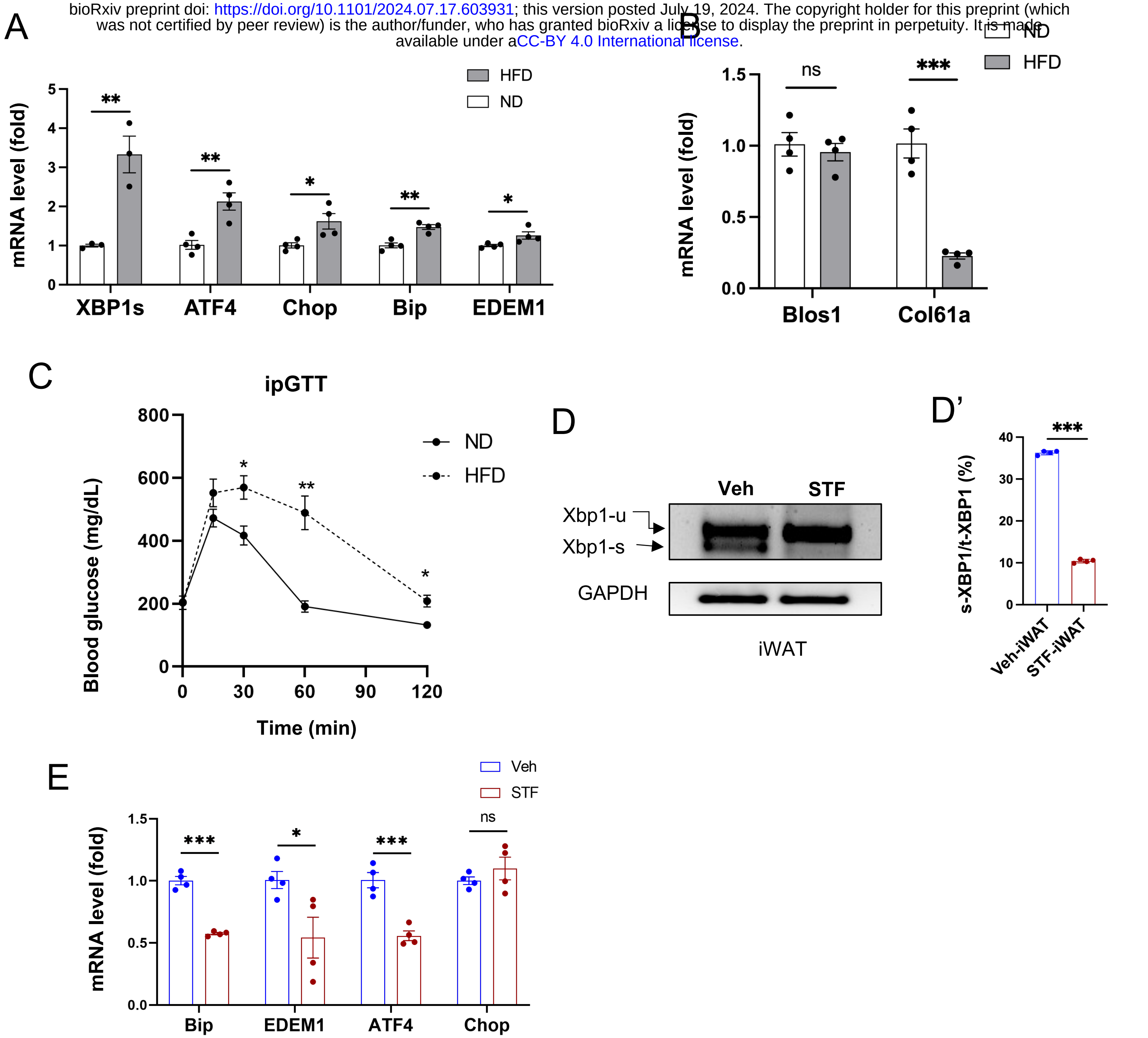

S Figure 1. ER stress and IRE1α activation in adipose tissues in DIO mice. A-B. mRNA levels of ER stress genes (A) and of IRE1α RIDD target genes (B) in the eWAT from mice fed ND (normal diet) and HFD for 14 weeks, assessed by qRT-PCR. The results are expressed as fold change and are representative of 3 independent experiments. C. Glucose tolerance test performed on mice on ND and HFD for 8 weeks. Blood glucose levels measured at indicated time points after intraperitoneal injection of glucose (1.5 g/kg body weight) following 6-h fasting. D-D'. XBP1 mRNA levels were analyzed from eWAT of DIO mice treated with vehicle or STF by RT-PCR and resolved by agarose gel electrophoresis. The full length (unspliced, XBP1-u) and spliced (XBP1-s) forms of XBP1 mRNA were indicated (D) and quantified (D'). GAPDH mRNA was used as an internal control. The data shown are representative of 3 independent experiments. E. mRNA levels for indicated genes were analyzed in eWAT of DIO mice treated with vehicle or STF by qRT-PCR. The results are expressed as fold change and are representative of 3 independent experiments. Data are expressed as mean ± SEM. $*P < 0.05$, $**P < 0.01$, and $***P < 0.001$.

Suppl. Fig. 1

bioRxiv preprint doi: https://doi.org/10.1101/2024.07.17.603931; this version posted July 19, 2024. The copyright holder for this preprint (which was not certified by peer review) is the author/funder, who has granted bioRxiv a license to display the preprint in perpetuity. It is made available under aCC-BY 4.0 International license.

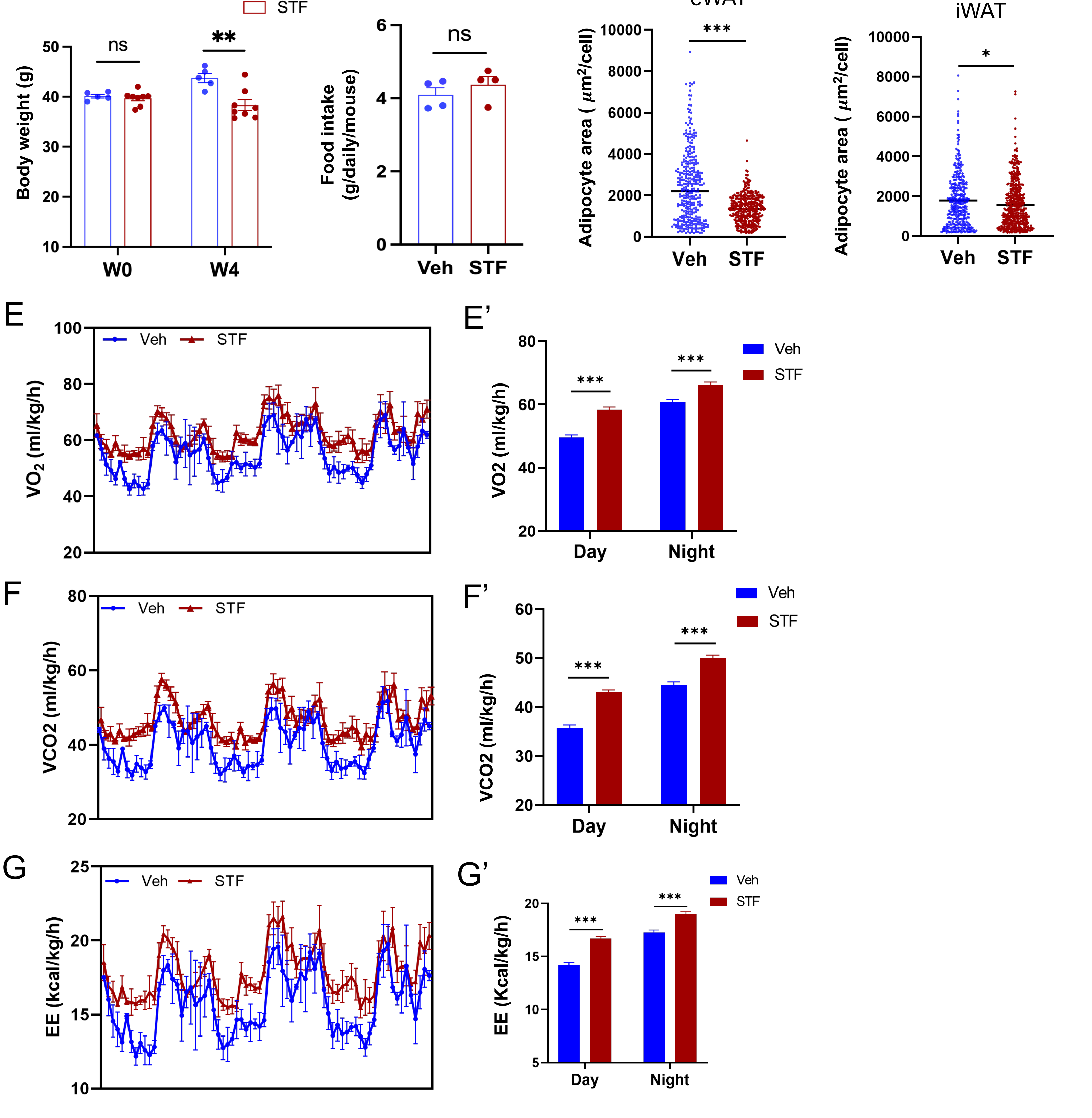

S Figure 2. STF protects against diet-induced obesity and promotes energy expenditure. A. Body weight of DIO mice treated with vehicle (n=5) or STF (n=8) before and after treatment for 4 weeks. B. Daily food intake from DIO mice treated with vehicle or STF, measured for three consecutive days. C-D. Adipocyte area per cell in the sections of eWAT (C) and iWAT (D). E-E'. Curves (E) and quantification (E') of oxygen consumption levels. F-F'. Curves (F) and quantification (F') of CO2 production levels. G-G'. Curves (G) and quantification (G') of energy expenditure levels calculated. For E-G', the data was normalized by body weight for analysis for group effect. Data were represented as mean ± SEM and analyzed using the unpaired two-tailed Student's t-test between two samples or ANOVA with multiple comparisons . ∗P < 0.05, ∗∗P < 0.01, and ∗∗∗P < 0.001.

Suppl. Fig. 2

bioRxiv preprint doi: https://doi.org/10.1101/2024.07.17.603931; this version posted July 19, 2024. The copyright holder for this preprint (which was not certified by peer review) is the author/funder, who has granted bioRxiv a license to display the preprint in perpetuity. It is made available under aCC-BY 4.0 International license.

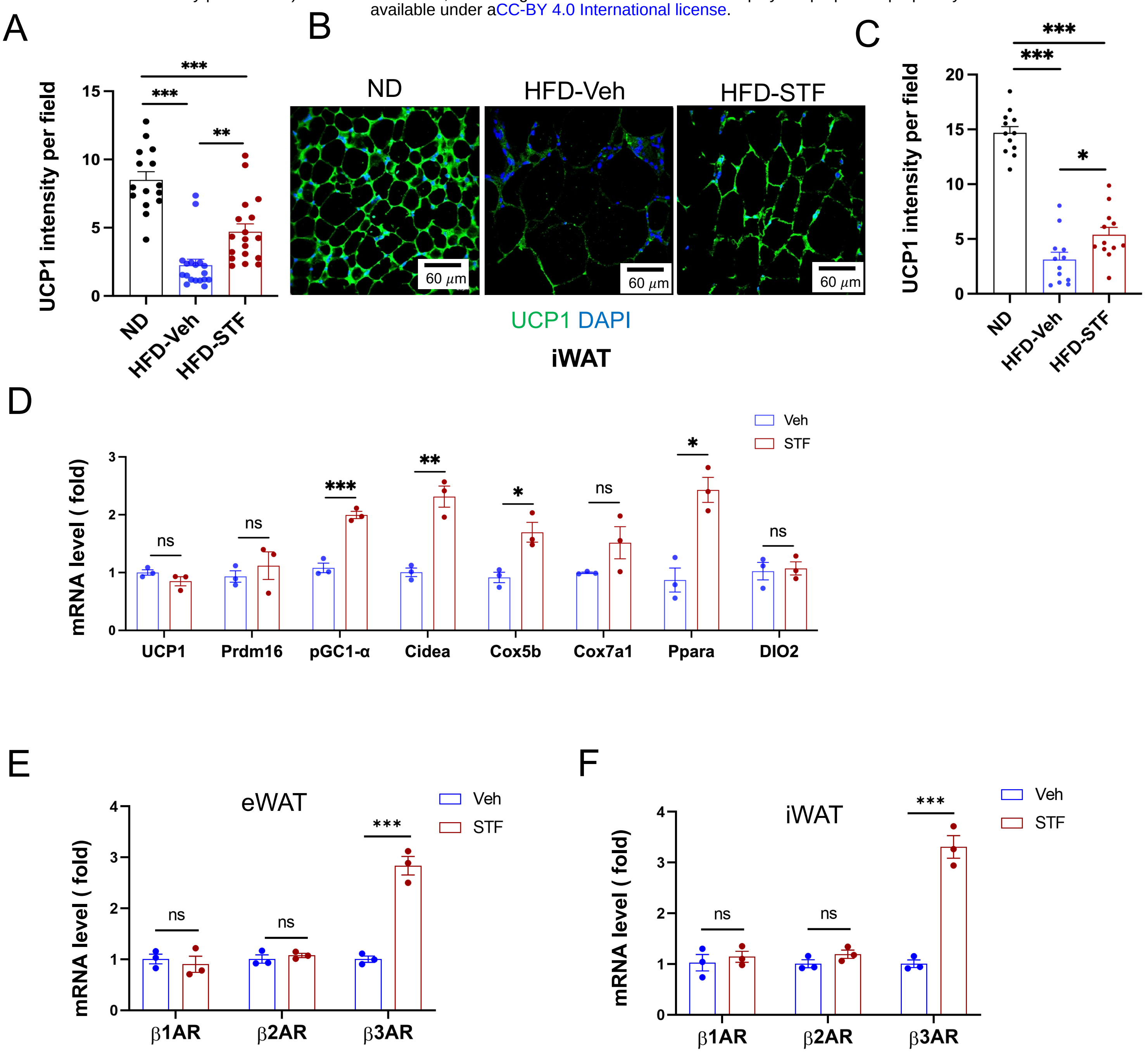

S Figure 3. STF promotes thermogenesis in WAT of DIO mice. A. Quantification of immune-signal intensity of UCP1 in eWAT sections from ND mice and DIO mice treated with vehicle or STF. B-C. UCP1 immunofluorescence staining of iWAT sections from ND-fed mice and DIO mice treated with vehicle or STF. Representative images of UCP1 immunofluorescence staining of iWAT (B) and quantification of immune-signal intensity of UCP1 (C). D. mRNA levels of thermogenic genes in iWAT from DIO mice treated with vehicle or STF, assessed by qRT-PCR. The results are expressed as fold change and are representative of 3 independent experiments. E-F. mRNA levels of β-adrenoceptor genes in eWAT (E) and iWAT (F) from DIO mice treated with vehicle or STF, assessed by qRT-PCR. The results are expressed as fold change and are representative of 3 independent experiments. Data are the mean ± SEM. $*P < 0.05$, $**P < 0.01$, and $***P < 0.001$.

Suppl. Fig. 3

bioRxiv preprint doi: https://doi.org/10.1101/2024.07.17.603931; this version posted July 19, 2024. The copyright holder for this preprint (which was not certified by peer review) is the author/funder, who has granted bioRxiv a license to display the preprint in perpetuity. It is made available under aCC-BY 4.0 International license.

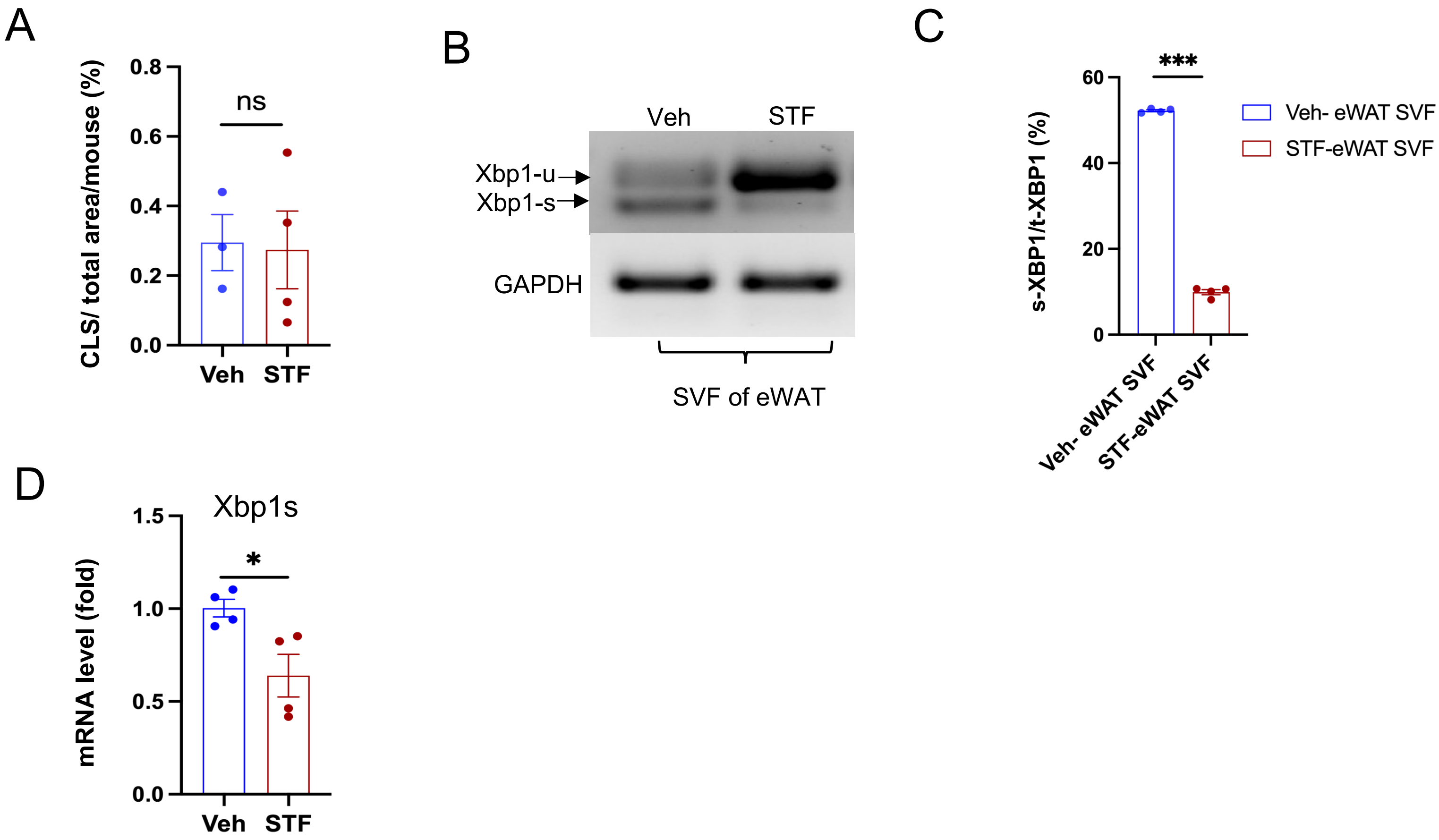

S Figure 4. STF inhibits IRE1α activity in the SVF of obese eWAT. A. Percentage of CLS area in iWAT sections from DIO mice treated with vehicle or STF. B-C. Splicing of XBP1 mRNA was analyzed from the SVFs of eWAT of DIO mice treated with vehicle or STF by RT-PCR and resolved by agarose gel electrophoresis. The full length (unspliced, XBP1-u) and spliced (XBP1-s) forms of XBP1 mRNA were indicated (B) and quantified with t-XBP1 as the sum of XBP1-u and XBP1-s (C). GAPDH mRNA was used as an internal control. The data shown are representative of 3 independent experiments. D. mRNA levels for indicated genes were analyzed in the SVFs of eWAT of DIO mice treated with vehicle or STF by qRT-PCR. The results are expressed as fold change and are representative of 3 independent experiments. Data are the mean ± SEM. $*P < 0.05$, $**P < 0.01$, and $***P < 0.001$.

bioRxiv preprint doi: https://doi.org/10.1101/2024.07.17.603931; this version posted July 19, 2024. The copyright holder for this preprint (which was not certified by peer review) is the author/funder, who has granted bioRxiv a license to display the preprint in perpetuity. It is made available under aCC-BY 4.0 International license.

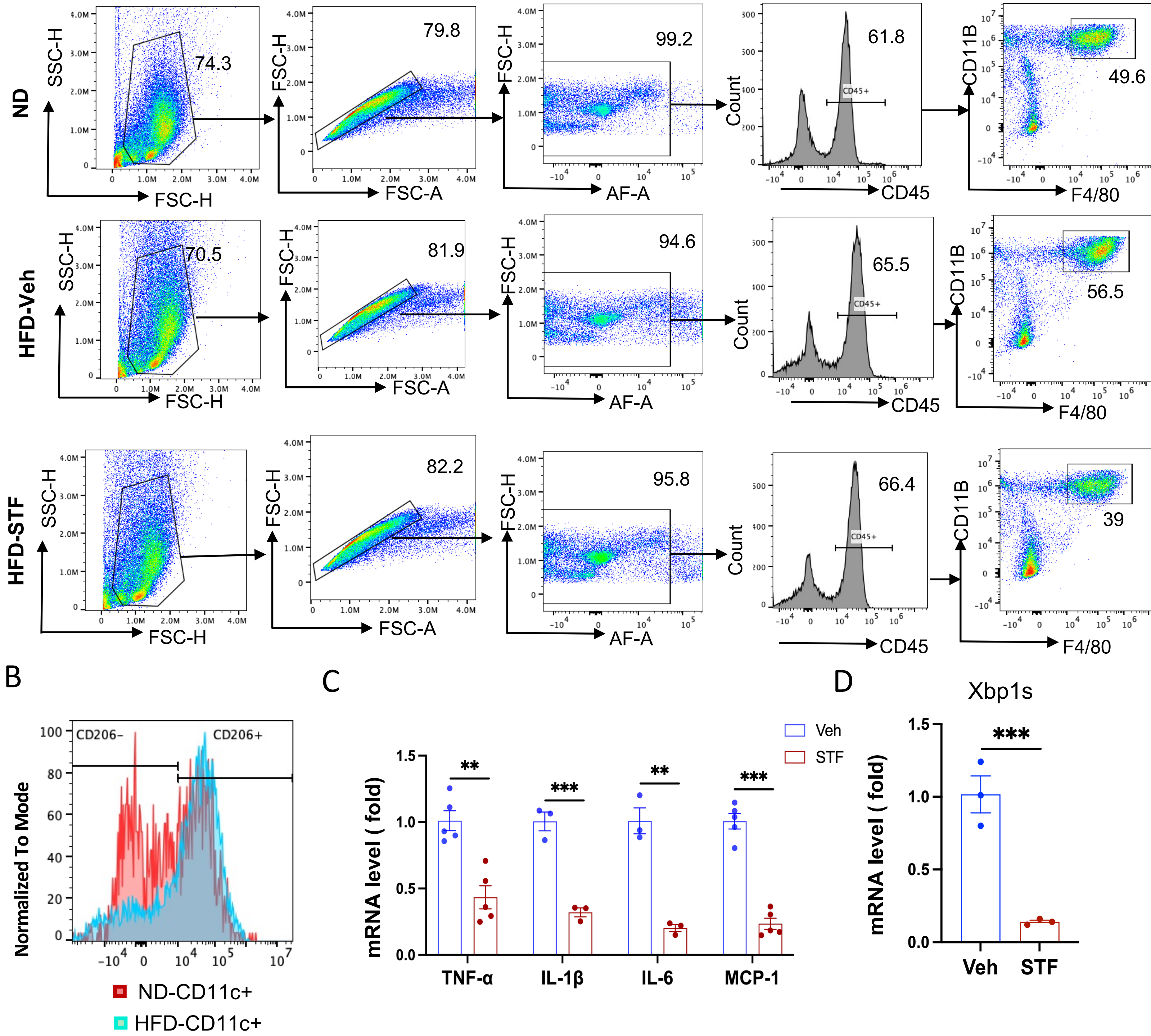

Suppl. Fig. 5

bioRxiv preprint doi: https://doi.org/10.1101/2024.07.17.603931; this version posted July 19, 2024. The copyright holder for this preprint (which was not certified by peer review) is the author/funder, who has granted bioRxiv a license to display the preprint in perpetuity. It is made available under aCC-BY 4.0 International license.

S Figure 5. STF suppresses obesity-driven ATM accumulation and adipose inflammation. A. Representative pseudocolor cell density flow cytometry plots showing the gating strategy used for analysis of macrophage subsets (F4/80^{+} CD11b^{+}) of SVFs of eWAT from ND-fed mice and DIO mice treated vehicle or STF, for figures shown in Fig. 5E. For Fig. 5F, G, I, J, Fig. 6H-N and Fig. 7G, markers were further gated immediately following the steps shown here. Numbers indicate percentage of gated cells. B. Flow cytometry analysis of CD206 signal in CD11c^{+} ATMs (F4/80^{+}CD11b^{+}CD11C^{+}) from eWAT SVFs of mice with an overlay of gated CD206 marker for ND and HFD. Complete gating path follows the strategy shown in A. C. mRNA levels of indicated proinflammatory cytokine/chemokine genes in the SVFs of iWAT of DIO mice treated with vehicle or STF, assessed by qRT-PCR. The results are expressed as fold change and are representative of 3 independent experiments. D. mRNA levels of splice XBP1 in the CD11C^{+} ATMs (F4/80^{+}CD11b^{+}CD11C^{+}) sorted from SVFs of eWAT of DIO mice treated with vehicle or STF, assessed by qRT-PCR. The results are expressed as fold change and are representative of 3 independent experiments. Data are the mean ± SEM. $*P < 0.05$, $**P < 0.01$, and $***P < 0.001$.

Suppl. Fig. 5 (cont.)

bioRxiv preprint doi: https://doi.org/10.1101/2024.07.17.603931; this version posted July 19, 2024. The copyright holder for this preprint (which was not certified by peer review) is the author/funder, who has granted bioRxiv a license to display the preprint in perpetuity. It is made available under aCC-BY 4.0 International license.

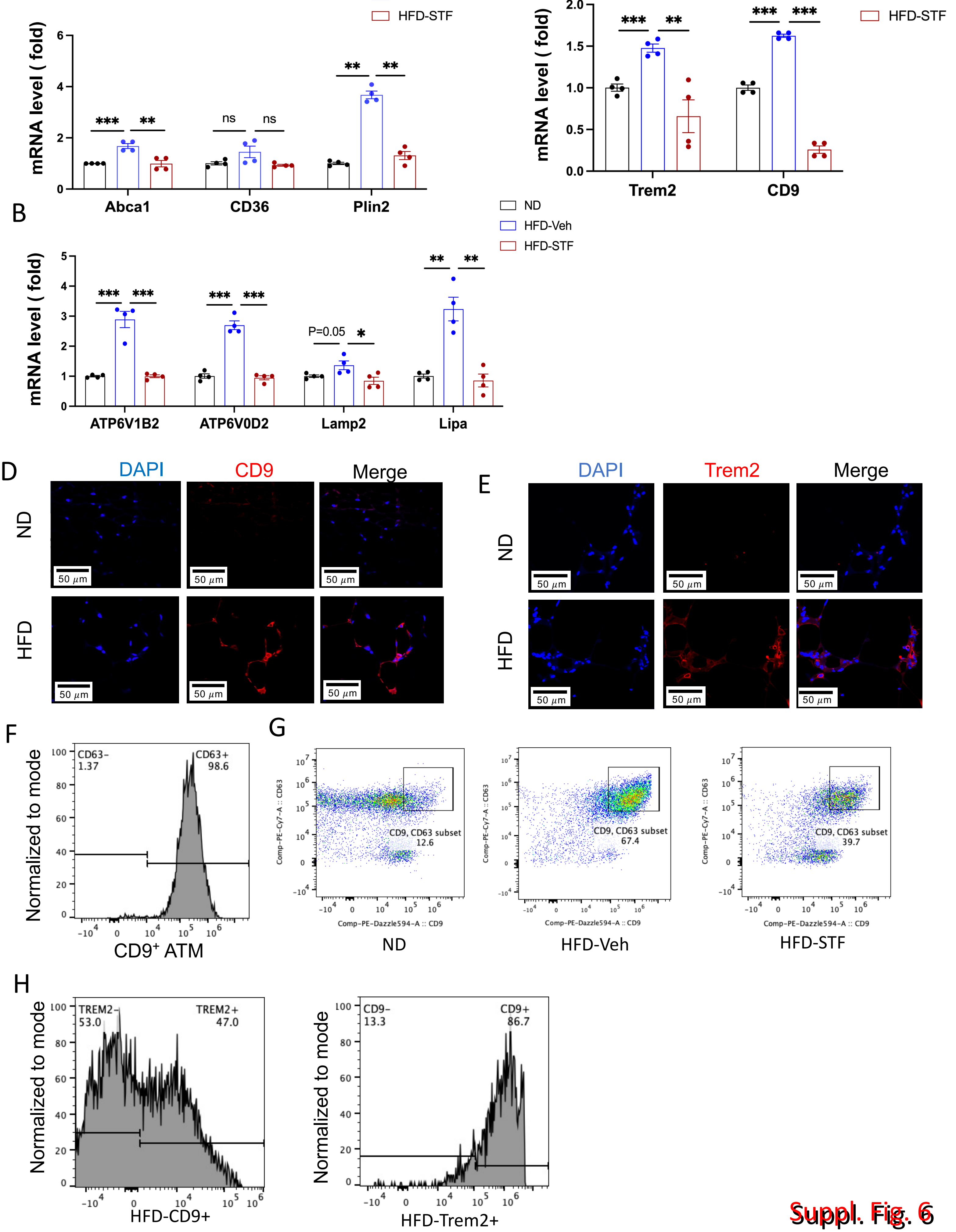

Suppl. Fig. 6

bioRxiv preprint doi: https://doi.org/10.1101/2024.07.17.603931; this version posted July 19, 2024. The copyright holder for this preprint (which was not certified by peer review) is the author/funder, who has granted bioRxiv a license to display the preprint in perpetuity. It is made available under aCC-BY 4.0 International license.

S Figure 6. STF suppresses metabolically active ATMs in DIO mice. A-C. mRNA levels of indicated genes in the SVFs of iWAT from ND, HFD-veh and HFD-STF mice, assessed by qRT-PCR. The results are expressed as fold change and are representative of 3 independent experiments. D. Representative images of immunofluorescent staining of CD9 (red) in the eWAT sections of ND- and HFD-fed mice. DAPI (blue) for nuclear staining. E. Representative images of immunofluorescent staining of Trem2 (red) in the eWAT sections of ND- and HFD-fed mice. DAPI (blue) for nuclear staining. F. Flow cytometry analysis of CD63 signal in $CD9^+$ ATMs from eWAT SVFs of DIO mice, gated against CD9 and CD63 antibodies sequentially following the gating strategy shown in Supplemental Figure 5A. G. Flow cytometry analysis of $CD9^+$ and $CD63^+$ ATMs from eWAT SVFs of ND, HFD-Veh and HFD-STF mice, gated against CD9 and Trem2 antibodies simultaneously following the gating strategy shown in Supplemental Figure 5A. H. Flow cytometry analyses of Trem2 signal in $CD9^+$ ATMs (left panel) and of CD9 signal in $Trem2^+$ ATMs (right panel) from eWAT SVFs of DIO mice, gated against CD9 and Trem2 antibodies sequentially (left panel) or against Trem2 and CD9 sequentially (right panel) following the gating strategy shown in Supplemental Figure 5A. Data are the mean ± SEM. $*P < 0.05$, $**P < 0.01$, and $***P < 0.001$.

Suppl. Fig. 6 cont.

bioRxiv preprint doi: https://doi.org/10.1101/2024.07.17.603931; this version posted July 19, 2024. The copyright holder for this preprint (which was not certified by peer review) is the author/funder, who has granted bioRxiv a license to display the preprint in perpetuity. It is made available under aCC-BY 4.0 International license.

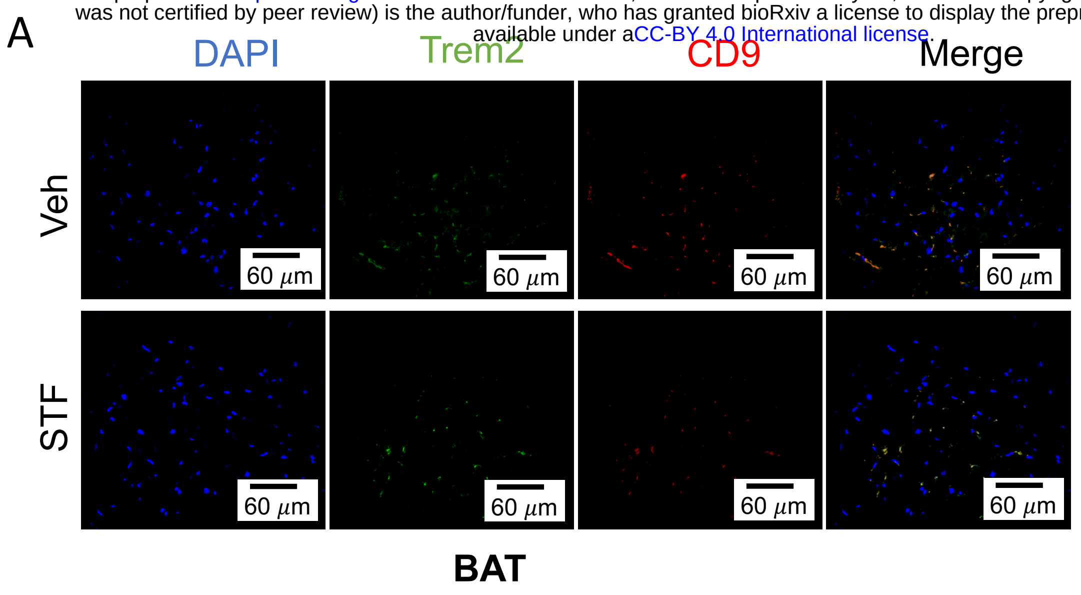

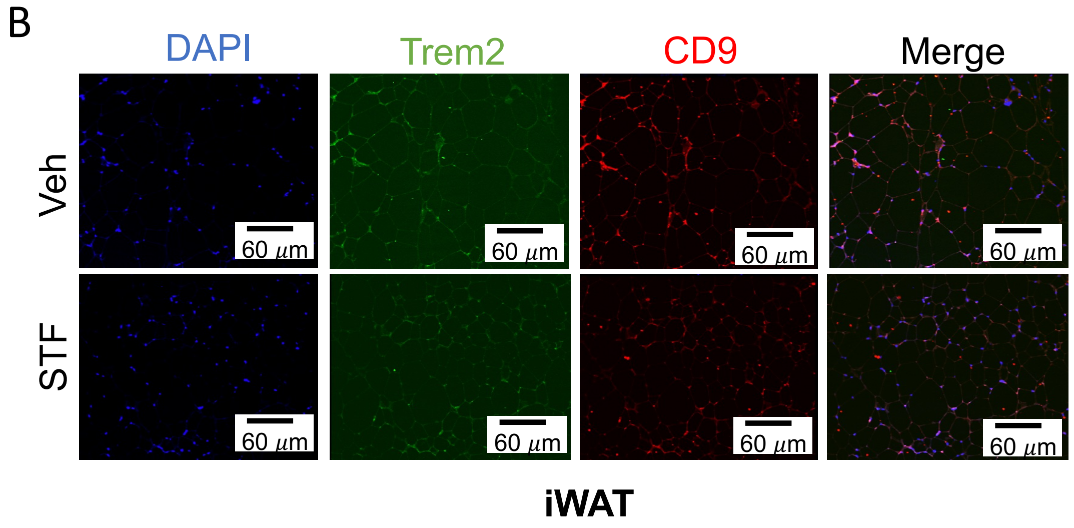

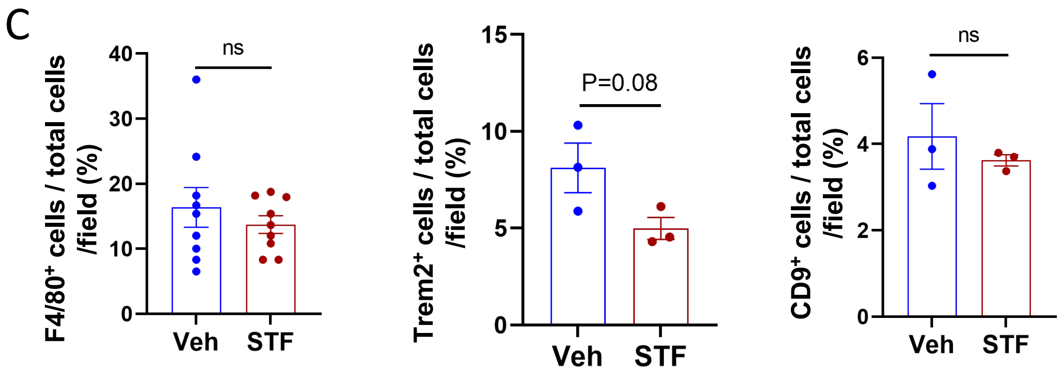

S Figure 7. STF suppresses CD9+ and Trem2+ ATMs in DIO mice. A. Representative images of immunofluorescent staining of CD9 (red) and Trem2 (green) in the BAT sections of HFD-fed mice treated with Veh or STF. DAPI (blue) for nuclear staining. B-C. Representative images of immunofluorescent staining in the iWAT sections of HFD-fed mice treated with Veh or STF (B) and quantification (C). CD9 (red), Trem2 (green), and DAPI for nuclear staining (blue).

Suppl. Fig. 7. New

bioRxiv preprint doi: https://doi.org/10.1101/2024.07.17.603931; this version posted July 19, 2024. The copyright holder for this preprint (which was not certified by peer review) is the author/funder, who has granted bioRxiv a license to display the preprint in perpetuity. It is made available under aCC-BY 4.0 International license.

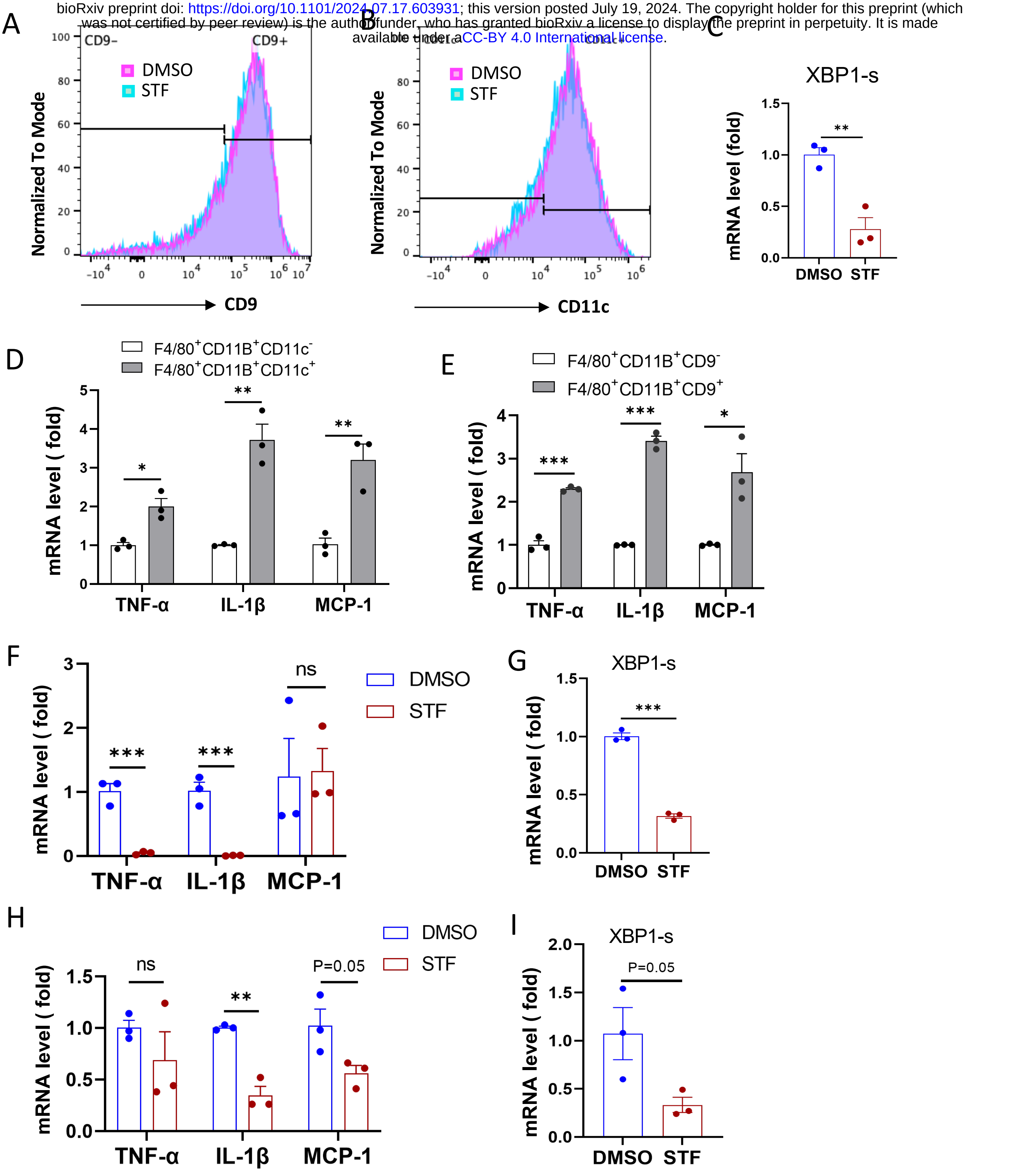

Suppl. Fig. 8. New

bioRxiv preprint doi: https://doi.org/10.1101/2024.07.17.603931; this version posted July 19, 2024. The copyright holder for this preprint (which was not certified by peer review) is the author/funder, who has granted bioRxiv a license to display the preprint in perpetuity. It is made available under aCC-BY 4.0 International license.

S Figure 8. STF suppresses pro-inflammatory cytokines genes expression in ATM in vitro. A-B. Flow cytometry analysis of ATMs from SVFs of the eWAT from 20-week HFD-fed mice. SVFs were cultured and treated with 0.01% DMSO control or STF (30 μM) for 20 h followed by staining and flow analysis, gated against CD9 antibody (A) or CD11C antibody (B). The gating path followed the one as Supplemental Figure 5A. C. mRNA levels of indicated genes in $CD11C^+$ ATMs sorted from SVFs cultured and treated with DMSO or STF, assessed by qRT-PCR. D-E. Comparison of mRNA levels of indicated proinflammatory genes between $CD11C^+$ and $CD11C^-$ ATMs (D) and between $CD9^+$ and $CD9^-$ ATMs (E), sorted from SVFs, assessed by qRT-PCR. F-G. mRNA levels of indicated proinflammatory genes (F) and spliced-XBP1 (G) in $CD9^-$ ATMs sorted from SVFs cultured and treated with DMSO or STF, assessed by qRT-PCR. F-G. mRNA levels of indicated proinflammatory genes (F) and spliced-XBP1 (G) in $CD11C^-$ ATMs sorted from SVFs cultured and treated with DMSO or STF, assessed by qRT-PCR. Data were expressed as mean ± SEM and analyzed using the unpaired two-tailed Student's t-test between two samples or ANOVA with multiple comparisons. $*P < 0.05$, $**P < 0.01$, and $***P < 0.001$..

Suppl. Fig. 8. New. Cont.

bioRxiv preprint doi: https://doi.org/10.1101/2024.07.17.603931; this version posted July 19, 2024. The copyright holder for this preprint (which was not certified by peer review) is the author/funder, who has granted bioRxiv a license to display the preprint in perpetuity. It is made available under aCC-BY 4.0 International license.

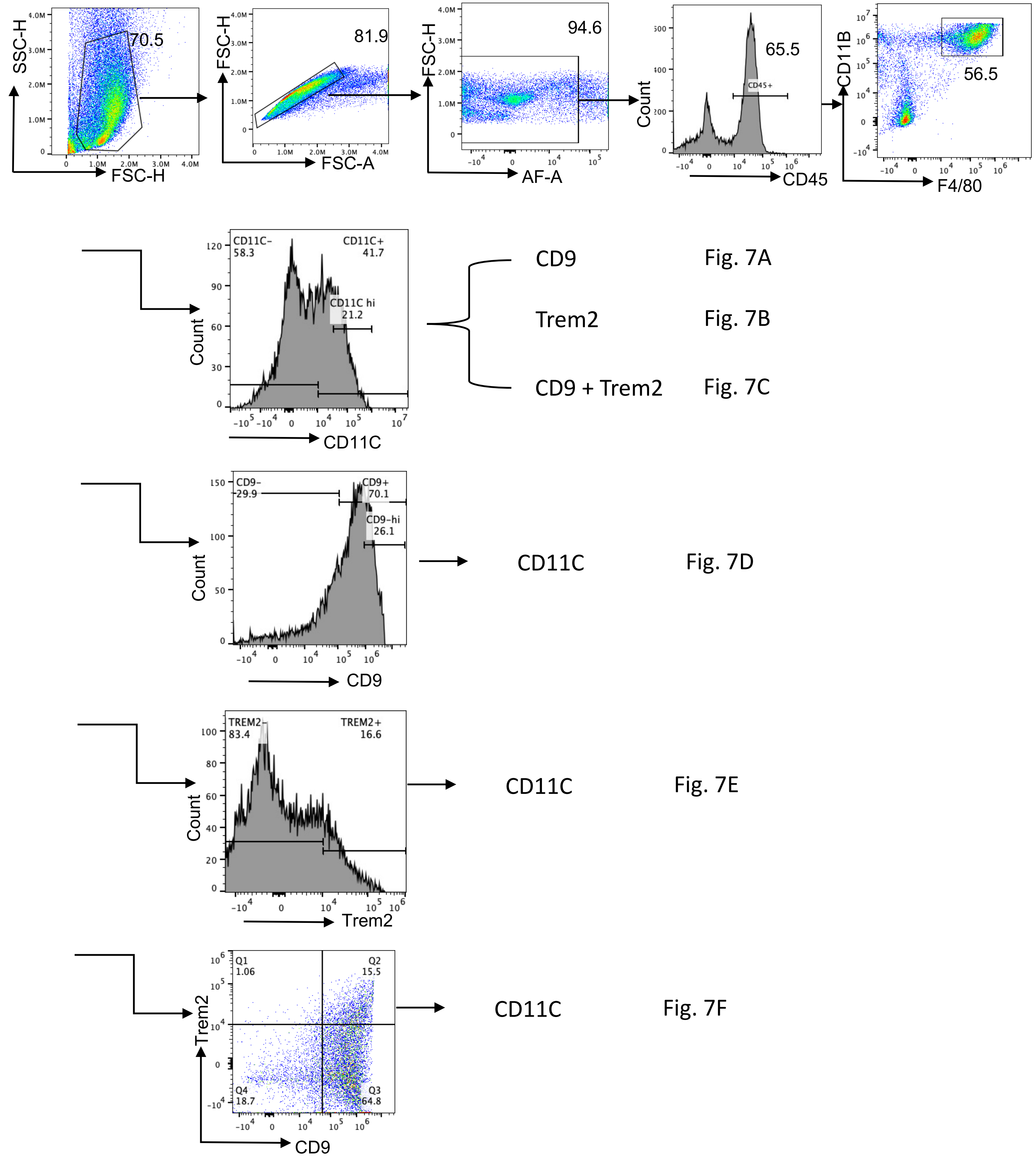

S Figure 9. Flow cytometry analysis strategy related to Figure 7.
Representative pseudocolor cell density flow cytometry plots showing the gating strategy used for analysis of subsets of ATMs in the eWATs from DIO mice with the total F4/80⁺ CD11b⁺ at the top row, followed by the steps specifically for Fig. 7A-F. Numbers indicate percentage of gated cells.

Suppl. Fig. 9

bioRxiv preprint doi: https://doi.org/10.1101/2024.07.17.603931; this version posted July 19, 2024. The copyright holder for this preprint (which was not certified by peer review) is the author/funder, who has granted bioRxiv a license to display the preprint in perpetuity. It is made available under aCC-BY 4.0 International license.

Table S1a

Table S1

F4/80$^+$CD11B$^+$CD11C$^+$CD206$^-$		
	Total cell#/eWAT/mouse	Cell#/g of eWAT/mouse
ND	0.12 ± 0.11 x10^4	0.25 ± 0.23 x10^4
HFD-Veh	1.43 ± 0.79 x10^5	0.74 ± 0.37 x10^5
HFD-STF	0.65 ± 0.43 x10^5	0.28 ± 0.17 x10^5
HFD-Veh/ND: Fold (P value)	118.61 (0.036)	30.27 (0.027)
HFD-STF/HFD-Veh: Fold (P value)	0.45 (0.20)	0.38 (0.12)

Table S1b

F4/80$^+$CD11B$^+$CD206$^-$CD11C$^-$		
	Total cell#/eWAT/mouse	Cell#/g of eWAT/mouse
ND	0.42 ± 0.42 x10^4	0.84 ± 0.83 x10^4
HFD-Veh	0.71 ± 0.13 x10^5	0.38 ± 0.038 x10^5
HFD-STF	1.30 ± 1.03 x10^5	0.56 ± 0.41 x10^5
HFD-Veh/ND: Fold (P value)	16.97 (0.038)	4.54 (0.084)
HFD-STF/HFD-Veh: Fold (P value)	1.83 (0.63)	1.46 (0.71)

Table S1c

F4-80$^-$CD11B$^-$		
	Total cell#/eWAT/mouse	Cell#/g of eWAT/mouse
HFD-Veh	7.04 ± 1.17 x10^5	3.78 ± 0.33 x10^5
HFD-STF	9.28 ± 1.57 x10^5	4.32 ± 0.20 x10^5
HFD-STF/HFD-Veh: Fold (P value)	1.32 (0.37)	1.14 (0.29)

Table S1. Effect of STF treatment on "M1-like" and "M2-like" ATMs in obesity. The total cell number and density of populations from CD11C$^+$CD206$^-$ ATMs in the eWATs of mice with ND, HFD-Veh, or HFD-STF (a), CD11C$^-$CD206$^-$ ATMs in the eWATs of mice with ND, HFD-Veh, or HFD-STF (b), and F4/80$^-$CD11B$^-$ ATMs in the eWATs of DIO mice treated with Veh or STF (c). Data in a-c were obtained from 2 batches of 4-5 mice each and are the mean ± SEM. $*P < 0.05$, $**P < 0.01$, and $***P < 0.001$

bioRxiv preprint doi: https://doi.org/10.1101/2024.07.17.603931; this version posted July 19, 2024. The copyright holder for this preprint (which was not certified by peer review) is the author/funder, who has granted bioRxiv a license to display the preprint in perpetuity. It is made available under aCC-BY 4.0 International license.

Table S2

Table S2

**F4-80⁺CD11B⁺CD9⁺CD63⁺**			
	Percentage	Total cell#/eWAT/mouse	Cell#/g of eWAT/mouse
ND	18.00 ± 5.40	0.38 ± 0.29 x10^4	0.87 ± 0.48 x10^4
HFD-Veh	65.90 ± 1.50	9.42 ± 0.78 x10^5	5.09 ± 0.011 x10^5
HFD-STF	49.35 ± 9.65	3.65 ± 0.08 x10^5	1.74 ± 0.25 x10^5
HFD-Veh/ND: Fold (P value)	3.66 (0.013)	245.23 (0.0069)	58.86 (9.59E-05)
HFD-STF/HFD-Veh: Fold (P value)	0.75 (0.23)	0.39 (0.018)	0.34 (0.0057)

Table S2. Characterization of and the effect of STF treatment on CD9$^+$ ATMs in DIO mice. The percentage, total cell number, and density of populations from CD9$^+$CD63$^+$ ATMs in the eWATs of mice with ND, HFD-Veh, or HFD-STF. Data were obtained from 2 batches of 4-5 mice each and are the mean ± SEM. $*P < 0.05$, $**P < 0.01$, and $***P < 0.001$

bioRxiv preprint doi: https://doi.org/10.1101/2024.07.17.603931; this version posted July 19, 2024. The copyright holder for this preprint (which was not certified by peer review) is the author/funder, who has granted bioRxiv a license to display the preprint in perpetuity. It is made available under aCC-BY 4.0 International license.

Supplemental Table 3
Primer sequences used in this work

Gene	Forward Primer	Reverse Primer
GAPDH	AAAGACTGGAGCCCCACACTCTAC	ATCCCGTATTTCACCTCTGCTTC
XBP1s	GAG TCC GCA GCA GGT	GTG TCA GAG TCC ATG
XBP1u	CGCAGACTGCTCGAGATAGA	CCACAAGGCCGTGAGTTTTC
Chop	GCAGCGACAGAGCCAGAATA	CAAGGTGAAAGGCAGGGACT
Bip	GTGTGTGAGACCAGAACCGT	GCAGTCAGGCAGGAGTCTTA
EDEM1	CAAGTGTGGGTACGCCACG	AAAGAAGCTCTCCATCCGGTC
Blos1	CAAGGAGCTGCAGGAGAAGA	CCAGGAGGGTGAAGTAAGAGG
Col61a	TGCTCAACATGAAGCAGACC	TTGAGGGAGAAAGCTCTGGA
ATF4	CCTATAAAGGCTTGCGGCCA	GCTGGATTTCGTGAAGAGCG
UCP1	CACCTTCCCCCTGGACACT	CCCTAGGACACCTTTATACCT
Prdm16	AGGAGGAGGAGAGAGATTCCG	GTCCGGGTCAGGTTCATACAT
pGC1-α	CCCTGCCATTGTTAAGACC	TGCTGCTGTTCCTGTTTTC
Cidea	ATCACAACTGGCCTGGTTACG	TACTACCCGGTGTCCATTTCT
Cox5b	TGCTACCTCCAAAGGCAGCTTC	CATCGCTGACTCTCGCCTTTGT
Cox7a1	CAGCGTCATGGTCAGTCTGT	AGAAAACCGTGTGGCAGAGA
Cox8b	GAACCATGAAGCCAACGACT	GCGAAGTTCACAGTGGTTCC
Ppara	TTCCCTGTGAACTGACGTTT	CCACCATGTTGGATGGATGTG
DIO2	AGAGTGGAGGCGCATGCT	GGCATCTAGGAGGAAGCTGTT
β1AR	CCGAAAGCAGGTGAATGCAA	AGCCAGTAAGCCATACTAAGCCACA
β2AR	CATTGATGTGTTGTGCGTCA	ACTCGGGCCTTATTCTTGGT
β3AR	CCTTCCGTCGTCTTCTGTGT	AGCCATCAAACCTGTTGAGC
TNF-α	CTGAACTTCGGGGTGATCGG	CTACGACGTGGGCTACAGG
IL-1β	TGCCACCTTTTGACAGTGATG	TGATGTGCTGCTGCGAGATT
IL-6	CACTTCACAAGTCGGAGGCT	CTGCAAGTGCATCATCGTTGT
MCP-1	AGGTCCCTGTCATGCTTCTG	TCTCCAGCCTACTCATTGGGA
Mgl1	CAGATCTGGGGCCGTCAAG	GGGAGGAAATGCATCTGGGT
IL-10	AGGCGCTGTCATCGATTTCT	ATGGCCTTGTAGACACCTTGG
F4/80	TCTGCTTCTGTACAGCCACG	CCTCAGAACCCACAGTGTCC
CD68	TGTTCAGCTCCAAGCCCAAA	GTACCGTCACAACCTCCCTG
Abca1	TAGCAGCACCGTGTCTTGTC	GCGTGTCACTTTCATGGTCG
